# Development of a solid dosage platform for the oral delivery of bilayer vesicles

**DOI:** 10.1016/j.ejps.2017.06.014

**Published:** 2017-10-15

**Authors:** Jitinder S. Wilkhu, Sarah E. McNeil, David E. Anderson, Marc Kirchmeier, Yvonne Perrie

**Affiliations:** a School of Life and Health Sciences, Aston University, Aston Triangle, Birmingham, B4 7ET, UK; b Variation Biotechnologies, 222 Third Street, Suite 2241, Cambridge, MA 02142, USA; c Strathclyde Institute of Pharmacy and Biomedical Sciences, University of Strathclyde, 161 Cathedral Street, Glasgow, G4 0RE, UK

**Keywords:** Liposomes, Niosomes, Vesicles, Oral disintegrating tablets, Oral vaccines

## Abstract

Within this work, we develop vesicles incorporating sub-unit antigens as solid dosage forms suitable for the oral delivery of vaccines. Using a combination of trehalose, dextran and mannitol, freeze-dried oral disintegrating tablets were formed which upon rehydration release bilayer vesicles incorporating antigen. Initial studies focused on the optimisation of the freeze-dry cycle and subsequently excipient content was optimised by testing tablet hardness, disintegration time and moisture content. The use of 10% mannitol and 10% dextran produced durable tablets which offered strong resistance to mechanical damage yet appropriate disintegration times and dispersed to release niosomes-entrapping antigen. From these studies, we have formulated a bilayer vesicle vaccine delivery system as rapid disintegrating tablets and capsules.

## Graphical abstract

**Figure:**
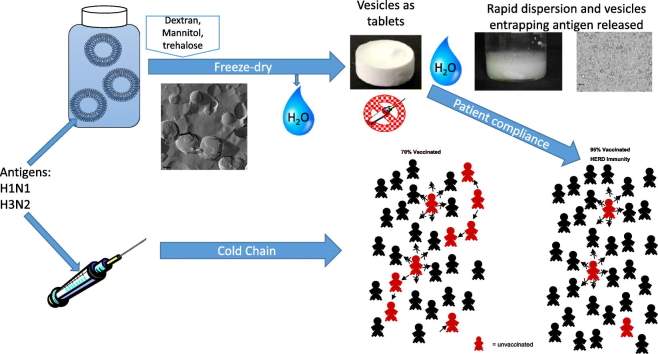

## 1 Introduction

The oral route as a means of drug delivery and immunisation offers a range of advantages including ease of administration, reduced need for trained personnel to administer vaccines and generally an increased convenience and compliance. Furthermore, in the case of vaccines, given that the mucosal sites are often the primary access point for human pathogens, oral vaccination can enhance mucosal immunity and promote strong resistance against many pathogens. In the development of oral vaccines, a range of delivery systems have been considered including liposomes and non-ionic surfactant vesicle carriers, such as niosomes or bilosomes. These systems are employed to encapsulate/associate vaccine antigens and thus provide protection and targeting within the gastro-intestinal tract. In previous studies from our group, we have shown that liposomes (Perrie et al., 2002) were able to protect and deliver DNA vaccines orally and non-ionic vesicles (with and without the addition of bile salts) were able to protect and deliver sub-unit antigens to within the target site of the Peyer's Patches (Wilkhu et al., 2013, Wilkhu et al., 2014). Furthermore, these bilayer vaccines incorporating recombinant HA were able to reduce median temperature differential change and promote a reduction in viral cell load in an influenza challenge study (Wilkhu et al., 2013).

In the development of an oral vaccine, generally the dosage form has been in a liquid format. However in terms of shelf stability, storage and distribution, vaccines in a liquid dosage form are not ideal and development of a stable solid dosage vaccine platform is required. Solid dosage forms such as tablets and capsules are the most commonly adopted oral delivery system, offering high patient compliance and easy storage. In addition, options such as orally disintegrating tablets are useful for paediatrics, geriatrics, patients who may struggle with swallowing (after stroke or renal failure patients) or patients with dysphasia (Lindgren and Janzon, 1991, Wilson et al., 1987, Gupta, 2010). Such solid dosage forms also offer a cost effective way to carry out bulk immunisation, as tablets can be distributed worldwide without the use of trained personnel.

To format liposomes into a dry format, freeze drying has been widely used as a standard method. Freeze drying of liposomes is used to prevent hydrolysis and physical degradation of the phospholipids within the vesicles during extended storage (Van Winden, 2003, Bridges and Taylor, 2001). However, the process of freezing and resultant dehydration of the formulation can exert stress onto the vesicles thus affecting the integrity of the vesicles; freezing may result in ice formation thus disrupting the bilayers and result in phase transition changes (Stark et al., 2010). Furthermore, upon dehydration, an increase in solute concentration can occur which may cause bilayer fractures, subsequently leading to vesicle aggregation, changes in vesicle size, and loss of entrapped antigen/material (Crowe et al., 1985, Crowe et al., 1986, Stark et al., 2010).

The associated problems with freeze drying of bilayer vesicles can be minimised by the inclusion of cryo- and lyoprotectants (which include disaccharide carbohydrate sugars) within the formulation prior to freeze drying. Protectants such as trehalose or sucrose are characterised by their non-eutectic nature, thus protecting the vesicles by forming an amorphous matrix around the vesicles. The addition of trehalose to a vesicle suspension inhibits vesicle fusion and aggregation during the freezing process (Abdelwahed et al., 2006, Van Winden et al., 1997). The mechanism of action of such cryoprotectants has been examined by Crowe et al. (1996) where they found that the sugar molecules are able to interact with the head groups of the phospholipids, thus, preventing membrane disruption by counteracting fusion (Crowe et al., 1996). In addition to using cryoprotectants to stablise the suspensions, other excipients have also been known to aid in the lyophilisation process, offering protection of the product. This includes bulking agents such as hydroxyethyl starch, trehalose, mannitol, lactose, and glycine. These are used when the concentration of product is low. Stabilisers such as sucrose, lactose, trehalose and mannitol can be used to offer protection through the freezing stages and isotonicity modifiers (e.g. mannitol, sucrose and glycerol) can be used to control isotonicity. As can be seen, a range of studies have considered the production of bilayer vesicles in a dried format; however, as noted by Tan et al. (2013), to aid their clinical translation, research into converting bilayer vesicles into convenient oral drug delivery systems is required. Therefore, the aim of this work was to exploit such excipients to formulate an oral vaccine solid dosage platform which, upon rehydration or ingestion, releases the vesicles containing the antigen. These vesicles should also maintain their integrity, potency and function as they would in a liquid dosage form.

## 2 Materials and methods

To form the vesicles, the surfactants monopalmitoyl glycerol (MPG; Larodan AG, Sweden), synthetic cholesterol (Chol), dicetyl phosphate (DCP) (Sigma-Aldrich, UK) were used as this formulation has previously been shown to act as a successful vaccine delivery system orally (Wilkhu et al., 2013, Wilkhu et al., 2014). The buffers were made up of sodium bicarbonate (Sigma-Aldrich, UK) at pH 7.6, where hydrochloric acid and sodium hydroxide (NaOH) (Sigma-Aldrich, UK) were used for pH adjustments. For the antigen, a recombinant H3N2 sub-unit protein (Immune Tech, USA) was used.

### 2.1 Preparation of vesicles for lyophilisation

A 5:4:1 M ratio of MPG, CHO and DCP was weighed and placed into a 10 mL glass beaker. 25 mM sodium bicarbonate (pH 7.6) formed the aqueous phase and was placed in a heated water bath for 10 min at 30–35 °C. Whilst the aqueous buffer was preheated, the beaker containing the lipids was placed into a hot oil bath (120–125 °C) and melted for 10 min with occasional mixing. The beaker containing the molten mixture was removed from the oil bath and the buffered stock solution was immediately added and homogenised at 8000 rpm at 30–35 °C. After homogenising for 10 min, the homogenisation speed was reduced to 4000 rpm to act as a mixer and dextran (Sigma Aldrich, UK) was added into the solution for a minute followed by mannitol (Sigma Aldrich, UK) for another minute of mixing. A 400 mM trehalose solution was prepared and then added at a 1:1 ratio of vesicle mixture: trehalose in a bijoux tube which formed the mould for the tablets and were then pre-frozen in the − 70 °C freezer until the freeze drying cycle was ready to begin.

## 3 Lyophilisation cycle

Lyophilisation was performed with the Virtis Advantage (Bio Pharma) freeze dryer. The freeze drying protocol was set for primary drying to occur at − 40 °C for 48 h with a secondary drying cycle set at 20 °C for a further 10 h with a condenser temperature set at − 75 °C.

### 3.1 Freeze fracture microscopy of vesicles

A 5 μL drop of each incubation mixture was placed on a ridged, gold specimen support or was sandwiched between two copper plates for fracture in a double replica device. Samples were frozen by rapid plunging into a constantly stirred mixture of propane:isopentane (3:1) cooled by liquid nitrogen. Fracture was performed on a Balzers BAF 400D apparatus at a temperature of − 110 °C. Replicas were floated free on distilled water and cleaned in 40% chromic acid. Images were then viewed using a Jenway transmission electron microscope.

### 3.2 TGA of freeze dried tablets

A small sample of the tablets were broken and the sample was placed onto the Perkin Elmer TGA apparatus, weighed and then analysed. The sample was heated to 110 °C and moisture content was determined as a % weight loss of the sample. All samples were repeated in triplicate to determine moisture content and degradation. All formulations were carried out using nitrogen and air as the purge gasses.

### 3.3 Mechanical strength

The mechanical properties (Hardness) of the tablets were analysed by a Tinius Olsen texture analyser (Hounsfield, UK) equipped with a 50 N load cell. The instrument was calibrated with standard weights and the tablets were placed individually on a platform. The hardness was expressed as the peak force (N) after a 2 mm penetration of a 5 mm diameter probe at velocity of 6 mm/min was applied. The average of five batches was taken as replicates.

### 3.4 Disintegration time

Disintegration time was measured using the USP apparatus (Erweka, ZT3) test for disintegration where 800 mL distilled water was kept at constant temperature of 37 °C. A basket with a wire mesh was raised up and down within this 800 mL media at an interval of 30 cycles/min. Samples were run individually and were tested in triplicate.

### 3.5 Determination of vesicle size and zeta potential

The vesicle size distribution was determined using laser diffraction on a sympatec 2005 (Helos/BF) cuvette analyser. 20 μL of the vesicle suspension was diluted into the cuvette with 40 mL double distilled water. The zeta potential was measured in 1.5 mL double distilled water at 25 °C on a Zeta Plus Brookhaven Instrument.

### 3.6 Labelling and quantification of Antigen

Initially the H3N2 antigen was incubated with the fluorescent flamma fluor FPR-648 (Bio Acts) marker and a conjugation buffer was added and left at 30 °C for 4 h to conjugate. After this period the unbound fluorescence was removed by centrifugation through an amicon P-10 centrifugal 10 kD MWCO filter tube. The remaining fluorescent antigen was made back to volume and then a spike of this was used per formulation. The H3N2 spike was placed into the aqueous phase prior to homogenisation. Initially a calibration curve was constructed on the same plate as the formulations and all conditions for all formulations was kept constant. Vesicles were prepared incorporating the fluorescent antigen and freeze dried. For quantification of antigen association, ultra-centrifugation of the formulations was required to isolate antigen entrapped vesicles, from non-incorporated antigen. To achieve this, freeze dried samples were rehydrated and 300 μL aliquots of sample were diluted in a Beckman 3.9 mL Polo-allomer tube and centrifuged at 354,000 ×* g* for 45 min at 4 °C. This was repeated and the antigen loaded vesicles were then re-suspended with 100–200 μL of appropriate buffer and transferred to a black microplate for reading fluorescence and antigen quantification.

### 3.7 Statistical analysis

The results within this study are given as the mean ± S.D. unless stated otherwise. Differences between results were analysed by ANOVA. A probability factor of < 0.05 (*p* < 0.05) was considered to represent statistically significant difference.

## 4 Results and discussion

### 4.1 Optimisation of Freeze drying cycle

Lyophilisation is a time-consuming and energy intensive process, thus optimisation of a cycle is vital (Hilleman and Hurni, 1982). The first step of freeze drying is the thermal treatment of the formulation i.e., pre-freezing, where the liquid suspension is cooled and ice crystals are formed. As previously mentioned, the presence of ice crystals may disrupt bilayers and lead to vesicle instability (Stark et al., 2010). In general, fast freezing results in smaller ice crystals being produced, compared to slow freezing which results in large crystals and pores. As a result, pre freezing of the liquid suspensions can be carried out by freezing at − 70 °C to overcome the issue of producing large crystals and larger pores.

**Fig. 1:**
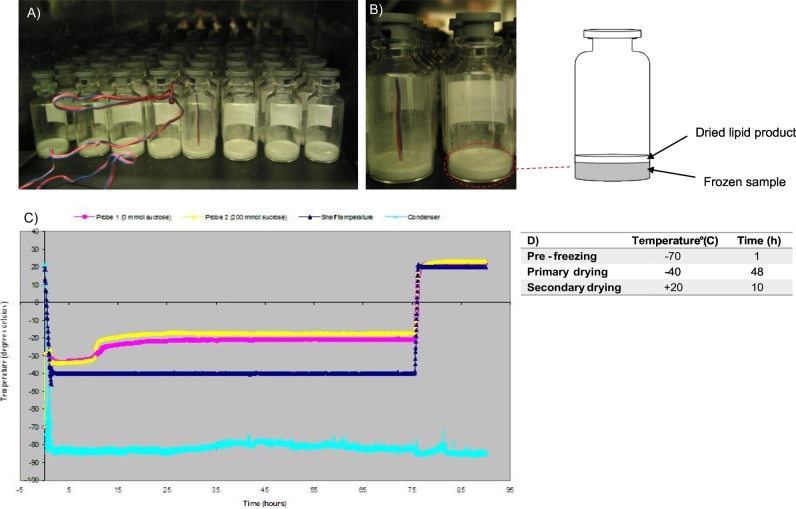
Samples undergoing lyophilisation where: A) Samples are in process during a freeze drying cycle. B) Sublimation taking place within a vial containing the vesicle formulation. Temperature probes are constantly monitoring internal temperature over the time period of the lyophilisation cycle. C) Key stages during a freeze drying cycle taken using a thermocouple placed inside vesicle suspension vials with and without sucrose. D) Final optimised parameters for lyophilisation of vesicles with condenser temperature set at − 75 °C.

Initially, the lyophilisation procedure for lipid based vesicles within this study was obtained from the development of a freeze drying protocol for TB-liposomal vaccines (Mohammed et al., 2006, Mohammed et al., 2007). Within these studies, it was found that liposomes, with the inclusion of a cryoprotectant, when pre frozen at − 70 °C followed by 10 h primary drying at − 50 °C with 24 h secondary drying at − 30 °C was a suitable protocol, as shown by the vesicle size after rehydration (Mohammed et al., 2006). The preparation of vesicles in this present study includes the use of significantly higher lipid concentrations within the formulations compared to the previous studies (Mohammed et al., 2006), hence the lyophilisation cycle was further optimised for drying times. This was carried out as presented in Fig. 1 where thermocouples were added to vials containing vesicles (Fig. 1 A and B) and Fig. 1B shows the sublimation front moving down through the sample. Fig. 1C presents this data where the shelf temperature was kept constant at − 40 °C and the change in temperature based on the thermocouples recorded the temperatures with and without a cryoprotectant. Fig. 1C shows that there was no deviation in temperature change between 30 and 75 h of primary drying, implying that the temperature within the lipid cake is constant thus a primary drying time between this time point can be adopted. Fig. 1C also demonstrates that the addition of a cryoprotectant (200 mM sucrose) allows primary drying to take place at a higher temperature (− 20 °C) as presented by the yellow line in the figure.

**Fig. 2:**
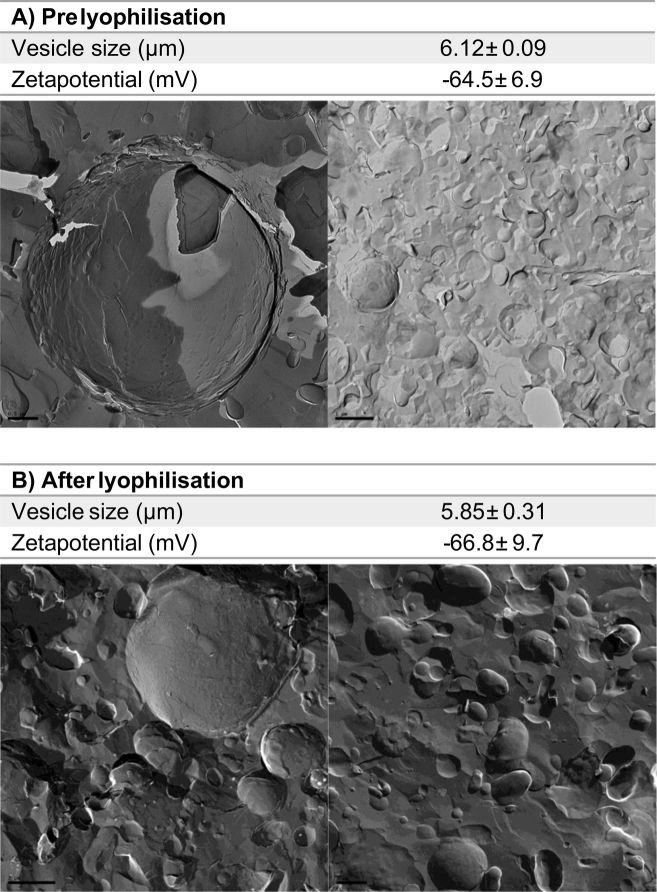
Vesicle characterisation data for vesicles prior to and after lyophilisation and imaged using freeze-fracture on Balzers apparatus at − 110 °C where images were then taken on a Jenway transmission electron microscope where: A) represents vesicles prior to freeze drying and B) rehydrated vesicles after freeze drying. Line bar represents 0.5 and 1 μm (A) and 2 and 1 μm (B).

Based on these results in Fig. 1, the final optimised protocol for the vesicles used primary drying for 48 h with a shelf temperature of − 40 °C. Size and zeta potential measurements, and freeze fracture images (Fig. 2) confirm the presence of bilayer vesicles prior to and after freeze drying, where Fig. 2A represents vesicles prior to freeze drying and Fig. 2B representing rehydrated vesicles after freeze drying. These results demonstrate that the size and zeta potential of the vesicles remaining unchanged after freeze-drying and re-suspension. Further chemical analysis was not undertaken of these formulations given the noisome components used within this study are widely recognised as stable and key attributes of concern for this work were the vesicle characteristics.

### 4.2 Formatting vesicles in oral dispersible tablets

To develop a freeze-dried tablet formulation of vesicles, mannitol was adopted as an additive as it is known to add bulking and stabilisation properties to the final freeze dried product (Seager, 1998). Mannitol is also water soluble and non-hygroscopic which provides a cooling sensation when in the mouth (Jeong et al., 2008). In addition, sucrose was compared with trehalose to determine any effects of protectant on the final product characteristics.

**Fig. 3:**
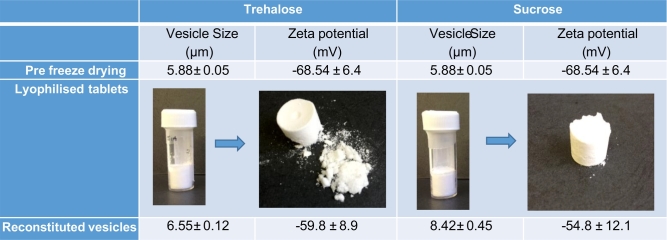
Final lyophilised tablets of vesicles containing 5% *w*/w mannitol representing appearance and handling protected with 200 mM final concentration of either A) trehalose and B) sucrose (*n* = 5).

**Fig. 4:**
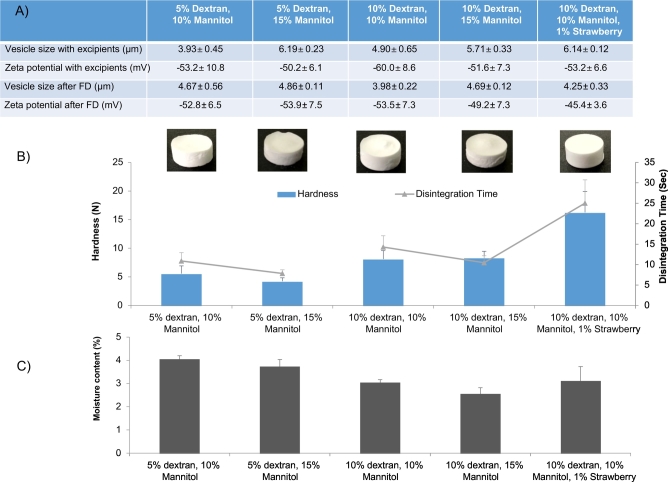
The result of varying mannitol and dextran ratios within the vesicle mixture where; A) outlines the vesicle characteristics, B) presents the appearance of the tablets, their hardness and disintegration time and C) their moisture content (*n* = 5).

Fig. 3 demonstrates the properties of the vesicles before freeze-drying and after reconstitution of the freeze-dried tablets containing 200 mM final concentration of either trehalose or sucrose. With the addition of sucrose as the cryoprotectant, a significant (*p* < 0.05) increase in vesicle size after rehydration was noted, with vesicles increasing to 8.42 ± 0.45 μm, compared to trehalose formulations where the vesicle size was retained (Fig. 3). In both cases, the anionic nature of the vesicles was retained (Fig. 3). Redispersion of the samples was carried out with 2 mL water and resuspension time was < 5 s (results not shown). The dispersion time of the tablets was rapid due to the matrix being prepared from water soluble sugars (sucrose/trehalose) thus allowing the moulded tablets to rapidly disintegrate. However, these moulded tablets had poor mechanical strength as demonstrated in Fig. 3; on removal of the tablets from the moulds, the handling was compromised. The highly porous nature of the freeze dried tablets results in their fragile properties. In general, freeze dried tablets such as Claritin® RediTabs® have highly porous inner structures which results in immediate disintegration and dissolving of the tablets upon the tongue (Fu et al., 2004). To increase the handling of the tablets, either direct compression can be used and/or binding agents can be added to the formulation. Therefore to improve the structural integrity and handling ability of the tablets in Fig. 3, the addition of different combinations of dextran and mannitol prior to lyophilisation was investigated (Fig. 4). The inclusion of a strawberry flavouring to one of the formulations was also considered. The formulations were tested for tablet hardness, disintegration time and resuspended to confirm vesicle size and zeta potential.

Prior to addition of excipients the vesicles were around 6 μm with a zeta potential of − 60 to − 70 mV (results not shown) in line with the results in Fig. 3. Upon addition of excipients, the vesicle size range decreases, which could be due to the dextran pulling down the volume mean diameter, and after lyophilisation and rehydration all of the formulations were approximately 4.5 μm (Fig. 4). The zeta potential values upon rehydration showed no significant differences between them and were around − 45 to − 55 mV and less negative than the initial readings prior to excipient addition (Fig. 4). Dextran and mannitol, are key excipients in controlling mechanical hardness and disintegration time (Chandrasekhar et al., 2009, Seager, 1998). Fig. 4B demonstrates that tablets containing 5% *w*/w dextran and 10% *w*/w mannitol have a tablet strength of 4 to 6 N and a disintegration time of approximately 10 s (Fig. 4) and increasing the mannitol content to 15% made no significant difference (*p* < 0.05). However, when the dextran content was increased to 10% both the tablet hardness and disintegration time increases and again, the difference in mannitol content (10 or 15%) made no significant difference. The addition of 1% *v*/v strawberry flavour to the formulation showed significantly (*p* < 0.05) higher tablet hardness (16 ± 1.4 N) and the tablets were prone to fracturing, resulting in the longest disintegration time. Studies have shown that the matrix of freeze dried tablets that consist of polymers such as gelatins, dextrans or alginates provide structural strength and saccharides such as mannitol or sorbitol provide crystallinity, hardness and elegance (Sastry et al., 2000). In the case of the freeze-dried vesicle formulations, all tablets produced are within the rapid disintegrating tablets class based on FDA criteria (where rapid disintegrating tablets should disintegrate in < 30 s (Mclaughlin et al., 2009, FDA, 2008)).

To determine moisture content within the formulations, thermogravimetric analysis studies (Fig. 4C) was carried based on a similar protocol to Mohammed et al. (2006) and all formulations were below the 5% moisture content threshold. The importance of moisture content within lyophilised tablets is due to the sensitivity of the excipients to residual moisture, which can impair long term stability and disintegration times. Furthermore, traces of moisture in a tablet may lead to longer term stability issues such as, friability, dissolution, microbial contamination and issues in the stability of the vesicles within the freeze-dried tablet. The moisture content results within this study are in line with Mohammed et al., (2006) where it was shown that cationic liposome vesicles, with the use of trehalose as the cryoprotectant, after freeze drying had residual moisture content levels < 5% (Mohammed et al., 2006). When correlating moisture content with tablet hardness, the results show that dextran content of 5% *w*/*v* results in higher moisture content (~ 4%) within the tablets and also reduced tablet hardness (4–6 N; Fig. 4). Water can affect the physical properties of freeze dried solids by inducing cake softening, cake collapse, crystallisation of amorphous solids, polymorphic conversions between crystalline structures and protein aggregation by increasing mobility within the solid (Costantino and Pikal, 2004). Studies by Nakabayashi et al., (1980) also demonstrated that the increase in moisture content within the lactose- corn starch tablets rapidly decreased the hardness of the tablets (Nakabayashi et al., 1980). Studies by Sugimoto et al. (2006) demonstrate that mannitol absorbed little moisture upon increase in relative humidity compared to excipients such as glucose or sorbitol (Sugimoto et al., 2006).

### 4.3 Formulation of vesicles entrapping antigen within solid dosage forms

The results in Fig. 4 show that the tablets incorporating freeze-dried vesicles can be formatted as rapid disintegrating tablets. However, the tablets produced still expose the vesicles to the external milieu of the stomach which can result in vesicle breakdown and/or loss of entrapped antigen if designed for the delivery of vaccines. Hence, as an alternative to tabletting, freeze dried vaccine formulations within capsules were also examined. Therefore vesicles were prepare containing the H3N2 antigen and loaded within capsule shells and compare the rehydration characteristics to a control tablet to ensure the capsule is not interfering with the vesicles.

**Fig. 5:**
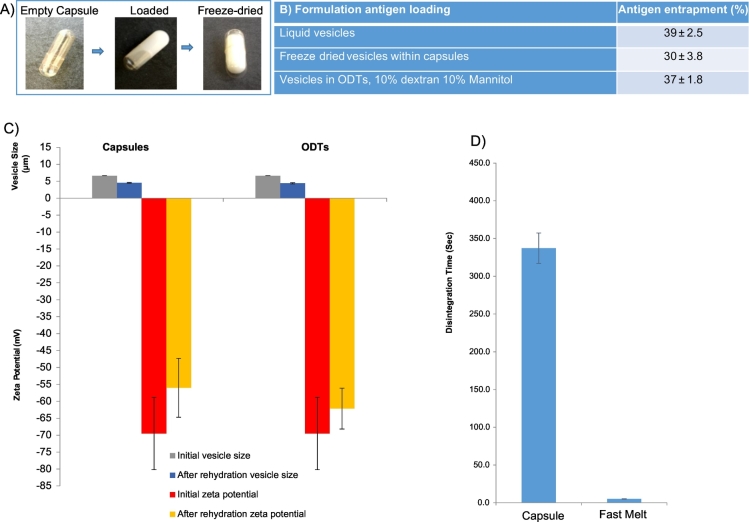
Vesicles containing antigen formatted as liquids, a freeze-dried tablet and ODTs where A) shows the various formulations B) shows antigen incorporation, C), outlines the vesicle characteristics and D) the disintegration time (*n* = 5).

The capsule formulations did not require the use of additives such as dextran or mannitol as the capsule shell provides structural integrity thus increasing handling and protection of the vesicular vaccines enclosed. The capsule shells are presented in Fig. 5A with a fill volume of 0.5 mL. Antigen loading, vesicle size distribution and surface charge was analysed prior to and after the freeze drying (Fig. 5). The initial vesicle size is comparable to previous formulations with an average vesicle size of 6–6.5 μm and a highly negative zeta potential (≥ 65 mV). After freeze-drying within the capsules, the contents were rehydrated with 0.5 mL ultrapure water, vortexed and then analysed for vesicle size and charge. The vesicles from the capsules reduced in size to an average of 4 to 5 μm with the zeta potential remaining highly negative (Fig. 5) which is within the size range suitable for uptake by the Peyer's patches (Tabata and Ikada, 1988, Ebel, 1990, Eldridge et al., 1990, Tabata et al., 1996).

Similarly, antigen loading within the vesicles was compared across all 3 dosage form platforms (liquid, freeze-dried capsules and ODT). Antigen loading was 30 to 40% of initial amount used, in line with the antigen loading of vesicles prior to freeze-drying (Fig. 5B). Fig. 5D represents the disintegration time of the capsule and the control tablet formulation within gastric media (pH 1.2). Results show that the tablets disintegrate within 5 s, compared to the capsules which have a disintegration/ release time of 5–6 min. In both cases, complete dispersion of the freeze-dried product and release of the niosomes was achieved. Capsules prepared by Jones et al. (2012), have shown that in humans in the fasted state, gelatin based capsules disintegrate within 7 ± 3 min compared to a fed state of 12 ± 3 min (Jones et al., 2012). The capsules prepared within this study offer this delayed release profile of 6 min and reduces the contact time of the vesicles and potentially any drugs/antigens prone to acid or enzymatic degradation within the stomach.

## 5 Conclusion

Within this study, we have produced an alternative solid dosage forms for the oral delivery of bilayer vesicles. Such systems can provide a more convenient and cost-effective delivery system for oral vaccine systems by helping the distribution and access to patients. Should improved targeting to the intestine be required, delayed release capsules could also be adopted to ensure the bilayer vesicle systems are not released until the small intestine. Furthermore, depending on the lipid composition and antigen selected, additional studies to assure the validity of the antigen may be required. However with the current bilayer vesicles, previous studies have shown these vesicles are stable within the GI tract, are taken up by the M cells and stimulate an immune response (Wilkhu et al., 2013), therefore the primary outcome of this study was to format the formulation in an easy to use solid dosage form.
